# Atrial Fibrillation and Cardiovascular Comorbidities, Survival and Mortality: A Real-Life Observational Study

**DOI:** 10.14740/cr324e

**Published:** 2014-02-27

**Authors:** Jose Luis Clua-Espuny, Anna Panisello-Tafalla, Carlos Lopez-Pablo, Inigo Lechuga-Duran, Ramon Bosch-Princep, Jorgina Lucas-Noll, Antonia Gonzalez-Henares, Lluisa Queralt-Tomas, Rosa Ripolles-Vicente, Cristina Calduch-Noll, Nuria Gonzalez-Rojas, Miguel Gallofre-Lopez

**Affiliations:** a Institut Catala de la Salut (ICS), Gerencia Territorial Terres de l’Ebre, Tortosa, Spain; b Department of Research, ICS Terres de l’Ebre, IDIAP Jordi Gol-IISPV, Tortosa, Spain; c Health Economics Department, Boehringer-Ingelheim, Sant Cugat del Valles, Barcelona, Spain; d Cerebral Vascular Disease’ Management Plan in Catalonia, Health Department Generalitat de Catalunya, Barcelona, Spain; e These authors contributed equally to this article

**Keywords:** Atrial fibrillation, Incidence, Cardiovascular complication, Survival, Mortality

## Abstract

**Background:**

Atrial fibrillation (AF) is the most common cardiac tachyarrhythmia encountered in clinical practice affecting up to 10% of the population over 60 years old and its prevalence rises with age. The main goals were to characterize the AF patient population after the initial diagnosis of AF and to determine overall survival.

**Methods:**

It is a real-life observational study of 269 subjects with an AF diagnosis over 60 years old randomly selected. The collected variables were: sociodemographic, cardiovascular complications/comorbidities (CVCs) included in the CHA_2_DS_2_-VASc and HAS_BLED scores, drug assigned as clinical treatment, mean range INR and CVCs and death dates (all-cause mortality). The survival curve and the risk of death were assessed using Kaplan-Meier survival curve and comparisons with log-rank.

**Results:**

The average following time was 6.2 ± 3.7 years (0.2-20.4). Eleven point five percent died. Sixty-five point four percent had some CVCs. There were no differences in the overall incidence of CVCs by gender. The survival probability was 0.86 ± DE 0.03 among men and 0.90 ± DE 0.04 among women without differences. Thirty-six point eight percent (95% CI: 30.8 - 42.7) were diagnosed vascular complications before AF diagnosis, being ischemic cardiopathy (24.2%) and ischemic stroke (23.2%) the most frequent. The mortality is higher (P < 0.036) among those who suffered ≥ 3 vascular complications and significantly lower among those treated with statins (P = 0.032). After AF diagnosis, the most frequent was the cardiac heart failure (46.7%), significantly higher among women (P = 0.037). The mortality is significantly lower in those treated with OAC (P = 0.003).

**Conclusions:**

AF is associated with ischemic heart disease, ischemic stroke and congestive heart failure, but the average mortality age is not different from the global population in Spain and Catalonia.

## Introduction

Atrial fibrillation (AF) is the most common cardiac tachyarrhythmia encountered in clinical practice affecting up to 10% [1] of the population over 60 years old and its prevalence rises with age. It is characterized by uncoordinated atrial activation that can lead to embolic complications and reduction in cardiac output resulting in significant morbidity, mortality and impaired quality of life. AF may cause stroke and heart failure and it is difficult to determine if cardiovascular events in patients with AF are attributable to the arrhythmia itself or if they are merely related to the comorbidities frequently associated with [2-4]. Modification of the general cardiovascular risk profile has largely remained the only option. Review of the literature suggests that AF is not an independent risk factor for mortality [5, 6] and the excess mortality observed in patients with chronic AF supports the independent role of the arrhythmia [7, 8]. On the other hand, if illnesses usually associated with AF are present, AF has a negative impact on outcome in terms of survival and morbidity with a 50-90% increase in the risk of death [9]. Thought the use of long-term anticoagulation with warfarin has been shown to offer an acceptable benefit/risk ratio. Furthermore, only those patients achieving an international normalized ratio (INR) control in excess of over 40% have significantly improved outcomes in terms of overall mortality [10] and comorbidities [11]. Knowledge on prediction and primary prevention of AF is still limited today. The main objectives were to characterize the AF patient population after the initial diagnosis of AF and to determine the overall survival.

## Methodology

Atrial Fibrillation Audit in Baix Ebre (AFABE) [1] study is a real-life observational of AF patient population, cross-sectional, multicenter study (22 primary care (PC) centers) in population over 60 years old attended by PC teams in Baix Ebre health area in Catalonia, north-eastern Spain in October 2012. The original aim of this study was to estimate the undiagnosed AF prevalence (AFp). According to this objective and taking into account the inhabitants of the area (82,000) over 60 years old population (20,733), the estimated AFp for the Spanish inhabitants (8.5%) [12] and assuming a 34% rate of non-diagnosis [13], we estimated that 600 patients in the Baix Ebre area would remain undiagnosed (2.9% of people over 60 years old). The sample size necessary to detect the percentage of undiagnosed patients was 1,029, given a 95% CI, a 1% margin of error and assuming a 2.9% undiagnosed AFp. In the end, 1,043 subjects were included in the study.

After ending the first part of the study and to get a representative sample of 269 people 60 years old diagnosed with AF, they were randomly selected among those who were visited at these health centers (90% of subjects) or at patient’s, old people’s, care or nursing homes (10%) with a documented AF diagnosis history in their electronic medical PC and hospital history and performed an additional interview. The number of selected patients in each PC center was proportional according to the number of people 60 years old assigned to each one of them. A patient was considered positive to AF when it could be demonstrated that they have a documented AF diagnosis history with at least an AF positive ECG [1]. Demographic and relevant clinical data such as previous cardiovascular events, cardiovascular-related diseases and other risk factors for AF were obtained. We performed a retrospective study of the cardiovascular conditions and associated prescriptions before and after the AF diagnosis using routine data collated until October 2012. In particular three datasets were used. The first was routine hospital inpatient history containing a range of information about diagnostic codes and destination on discharge attended by the referral specialties. The second was a hematology laboratory dataset containing details of all INR monitoring test results of those patients undertaken in hospital service. The third dataset was routine PC history containing a range of information about details of acute episodes using the ICD-10 classification, treatments, all INR monitoring test results when the patient was undertaken in PC and details of deaths. The information of the study’s variables was collected electronically in MSAccess program specifically created for this study. The professionals in charge of the collection of information and review of the clinical electronic histories were two medical specialists in familiar and community medicine specially trained.

The collected variables were:

1) Patient identification code: individualized number TIS (individual sanitary card used in Catalonia).

2) Socio-demographic information: age, gender, living place. The centers were classified as rural if they were located in villages with < 1,000 inhabitants, semi-urban for those located in towns and villages with 1,000-10,000 inhabitants and urban for towns with >10,000 inhabitants.

3) Cardiovascular information: registered cardiovascular complications/comorbidities (CVCs) included in the CHA_2_DS_2_-VASc [14, 15] and HAS_BLED [16, 17] scores and we considered them as “previous” when they had been diagnosed and registered at least one month before the AF diagnosis; and as “later” when they had been diagnosed and registered simultaneously or after FA diagnosis; what sort of AF [1] and where the diagnosis AF was confirmed: PC or hospital service (referred to emergency or cardiology). This study focuses upon patient with CHA_2_DS_2_-VASc ≥ 2 as it is in these patients who are considered eligible for anticoagulation treatment with warfarin.

4) Pharmacological information: drugs assigned as clinical treatment for AF: antiarrhythmic received as a rhythm control strategy (class I/class III) with or without rate control strategy (class II/class IV, digital); and/or antithrombotic treatment: with vitamin K antagonist or/and oral anticoagulant (OAC) therapy; and/or angiotensin-converting enzyme (ACE) inhibitors; and/or statins treatment.

5) Diagnosis’ dates: AF, CVCs and death dates (all-cause mortality) are those registered in their electronic medical PC. All the diagnostics were defined in the patient dataset using the ICD-10 classification. Given it was a retrospective study of confirmed AF, it has not been collected cases with a changed diagnosis of AF or with not confirmed AF.

6) INR control: It was estimated the percentage time in therapeutic international normalized ratio range (TTR) that an individual was within the therapeutic INR range (2-3) achieved among each patient who received prescriptions for warfarin and had five or more INR values within the laboratory dataset. We calculated TTR (between 0% and 100%) using Rosendaal’s method [18] which uses linear interpolation to assign an INR value to each day between two successive observed INR values. We calculated TTR for each patient. Warfarin is the anticoagulant therapy of choice in Catalonia for patients with AF who are in risk of stroke. The patients were stratified according to their proportion of time in range.

### Statistical analysis

In the descriptive analysis, data for categorical variables are expressed as number of cases and percentage, and data for continuous variables are expressed as mean with its standard deviation. Categorical variables were compared using a χ^2^ test or Fisher’s exact test as required. Continuous variables were compared using Student’s t test or Mann-Whitney test depending on the normality distribution assumption. An usual distribution was checked by the Shapiro-Wilk test. The survival curve and the risk of death were assessed using univariate and multivariate analyses, Kaplan-Meier survival curve and comparisons with log-rank. Factors that were independently associated in the univariate analysis, being at least marginally significant (P ≤ 0.1), were included using a backward step-wise strategy. A multivariate logistic regression analysis was performed to find associated risk factors to survival (all-cause mortality) of the population with AF. A P-value of less than 0.05 was considered to indicate statistical significance. The analysis was carried out with the SPSS statistical software package (version 19). Cox regressions models were constructed to model the time to next stroke and death controlling for age, sex and CHA_2_DS_2_-VASc score.

## Results

**Figure 1:**
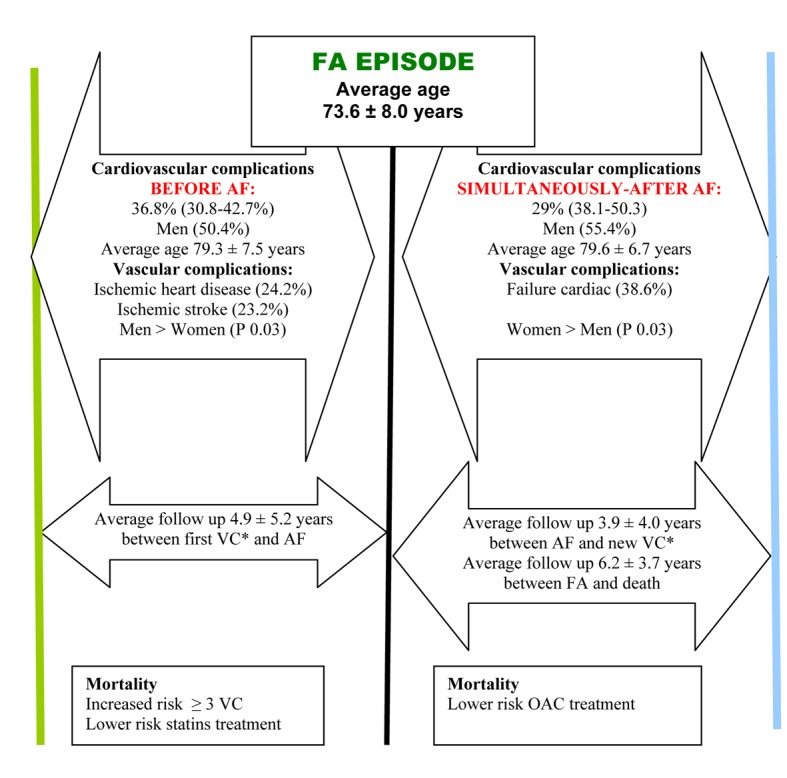
Longitudinal relationship between cardiovascular complications and atrial fibrillation. *Vascular complication.

We could sketch the temporal progression around the AF and its CVCs to one described in the Figure 1.

The basal characteristics of the AFABE study are showed in Table 1. The randomly selected sample was 269 AF cases registered in their clinical history. Men (50.2%) (133 women and 136 men). The patients with AF are older and their CHA_2_DS_2_-VASc score is higher than among general population. Three-fourth were ≥ 75 years. The average age at the diagnosis of AF was 73.65 ± DE: 8.0 years. The average following time was 6.2 ± 3.7 years (0.2-20.4). Eleven point seven percent died (45.1% women and 54.8% men; P = 0.665).

**Table 1:** Subjects Characteristics and Cardiovascular Risk Factors Prevalence in AF Affected Patients

Variables	General population without AF	Population with AF	P
N	1,043	269	
Women (%)	52.9	49.8	0.398
Mean age (years)	72.44 ± 7.59	78.7 ± 7.30	< 0.001
Type of population (%)			
Rural	16.4	16.2	1
Semi-urban	37.5	36.9	0.91
Urban	46.1	46.9	0.88
Age groups (%)			
< 65	15.7	3.7	< 0.001
65-74	40.9	21.4	< 0.001
≥ 75	43.3	74.9	< 0.001
Hypertension (%)	66	70.8	0.1468
Diabetes mellitus (%)	27	26.6	0.938
CHA_2_DS_2_-VASc score (%)			
0			
1	2.3	0 .7	0.16
2	10.7	5.2	0.008
3	24.2	13.3	< 0.001
4	28.9	19.6	0.008
5	21.4	28.0	0.025
6	8.3	15.9	< 0.001
7	2.2	12.2	< 0.001
8	1.5	3.3	0.096
CHA_2_DS_2_-VASc ≥ 2 (%)	85.9	95.6	0.004

The AF is associated with significant cardiovascular morbidity, primarily caused by ischemic stroke, stroke and cardiac heart failure (CHF) with the requirement for chronic use of medication (Table 2). The more usual way in the AF diagnosis was a first contact in PC (68.3%). The 56.3% of those were referred to hospital emergency service. Eventually the 56.0% (95% CI: 47.2 - 64.4) will be followed by cardiology service and the 41.8% (95% CI: 33.0 - 50.5) just by PC service. There are not differences in their clinical complexity but the patient group followed by the cardiologist is younger (P = 0.005), less treated with OAC (P = 0.003) and more prevalence of paroxysmal AF (P = 0.006). In reference to presence of CVC and its longitudinal relationship with the diagnosis AF, in 34.2% of patients, there have never been neither before nor after diagnosis AF. The percentage of patients with a score CHA_2_DS_2_-VASc ≥ 2 was 95.6%.

**Table 2:** Percentage of Specific Treatments

	Overall group (%)	CHA_2_DS_2_-VASc ≥ 2 (%)	Lone FA (%)
Rate control treatment	54.6	38.7	51.08
Antiarrhythmic treatment	33.0	19.7	36.7
Oral anticoagulant treatment	77.7	53.3	78.2
Antiplatelet therapy	19.7	18.2	21.7
ACE-inhibitors treatment	60.9	46.2	56.5
Statins treatment	49.0	35.5	38.0

**Figure 2:**
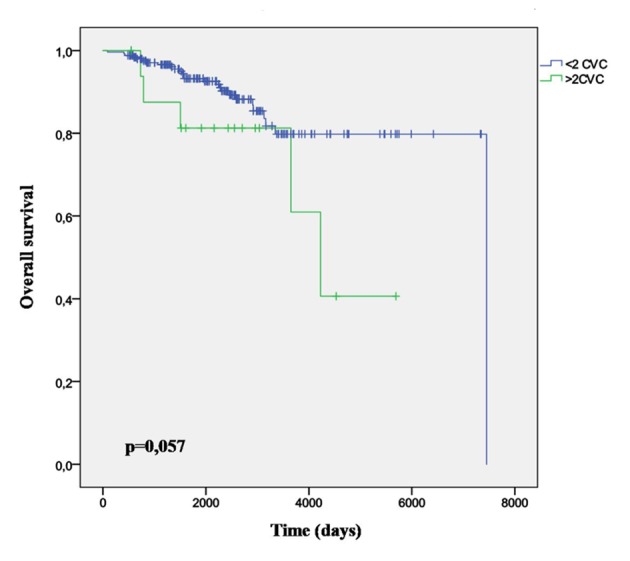
Survival curve in patients with > 2 diagnosed CVC.

Thirty-six point eight percent (95% CI: 30.8 - 42.7) have been diagnosed with some vascular complication before knowing the AF. Almost half of the overall vascular complications are ischemic cardiopathy (24.2%) and the ischemic stroke (23.2%). The ischemic cardiopathy incidence is significantly higher among men (P = 0.031), while the ischemic stroke incidence is similar among men and women (P = 0.612). There were no differences in the overall incidence of CVCs by gender. The 64.6% of these will be diagnosed of new CVC after or simultaneously to their AF diagnosis. The mortality according to the number of diagnosed vascular complications is higher (P = 0.036) among those who have been diagnosed with ≥ 3 vascular complications before the AF (Fig. 2). On the other hand, the mortality is significantly lower among those who were treated with statins (P = 0.032).

**Figure 3:**
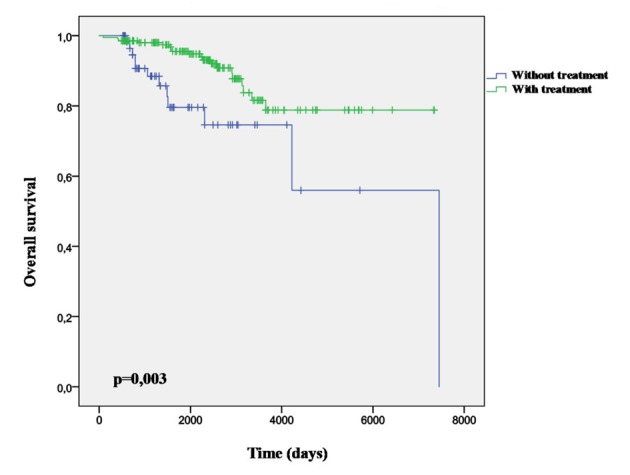
Survival curve and treatment with oral anticoagulant agents.

Twenty-eight point six percent (95% CI: 38.1 - 50.3) were diagnosed some new vascular complications simultaneously or after AF. The most frequent vascular complication was the CHF (46.7%) which had an incidence significantly higher among women (P = 0.037). At the end of the study, the survival chance with a diagnosis of CHF (0.69 ± DE 0.09) is lower than when there is no CHF present (0.96 ± DE 0.01). The mortality is significantly (Fig. 3) lower in those treated with OAC (P = 0.003) versus antiplatelet treatment. There were not differences in the overall incidence of CVCs by gender.

**Figure 4:**
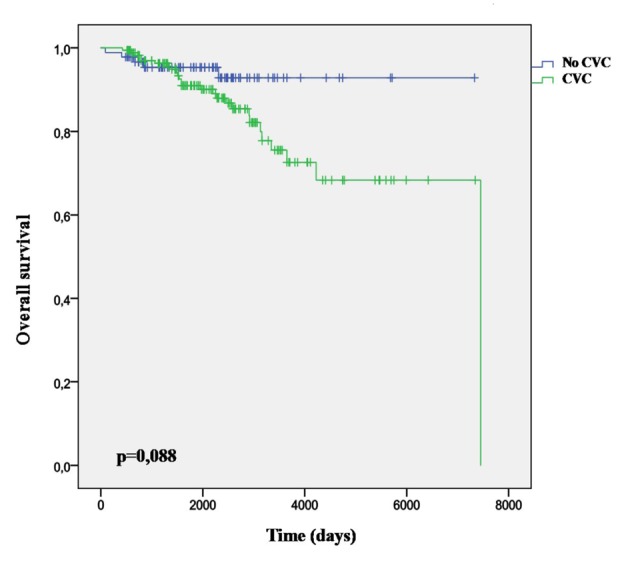
Survival curve in AF patients without CVC.

Thirty-four point two percent (95% CI: 28.3 - 40.0) were not diagnosed CVC through the following time, so considering the overall AF, around 3.4% out of patients with AF older than 60 years are going to be free of CVCs. The average age of this group was 77.6 years DE 7.77 significantly lower (P = 0.031) than those with CVCs diagnosed in any time along their evolution. Their survival curve was significantly better than the remaining (Fig. 4).

According to different sorts of treatments, there were only significant differences in mortality between the group with/without statins in the patients with CVCs before AF; the group with/without oral anticoagulant in the patients without CVCs though the average age (78.4 versus 80.7) of the patients treated is lower (P = 0.032). Sixty-seven point five percent of patients have TTR ≥ 60%. The mean TTR was 57.3% and its level has neither significant related differences with scores CHA_2_DS_2_-VASc (P = 0.292) nor mortality percentage (P = 0.851). We have not found different survival curves according to the mean time in TTR.

**Figure 5:**
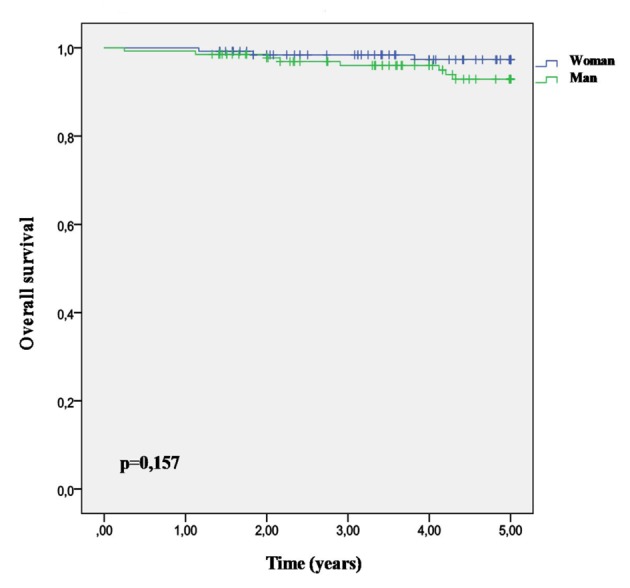
Survival curve to 5 years after AF diagnosis.

The mean age to death in patients with AF was 82.08 DE 4.86 years (74 - 94). The average duration of the following was 6.23 ± DE 3.65 years in the alive group and 5.81 ± DE 4.41 years in the death group without differences (P = 0.608). The survival probabilities were 0.92 ± DE 0.03 at 5 years, 0.80 ± DE 0.07 at 10 years, and 0.86 ± DE 0.03 among men and 0.90 ± DE 0.04 among women without differences (Fig. 5). The accumulated proportion of deaths in women is higher than that among the men (P = 0.005) in the first period of 5 years after the AF diagnosis, while among the men it is higher (P = 0.005) from the fifth to tenth year after AF diagnosis (Table 3).

**Table 3:** Accumulated Mortality by Gender and Period From AF Diagnosis

	N	Mortality 1 year	Mortality 5 years	Mortality 10 years	Total
Women	130	-	12/14 85.7% (95% IC)	2/14 14.2% (95% IC)	14 (10.7%)
Men	135	1/17 (5.8%)	5/17 29.4% (95% IC)	12/17 70.5% (95% IC)	17 (12.6%)
Total	265		17/31 (54.8%)	21/23	31 (11.7%)
P value			0.157	0.380	0.562

In the group of patients with score CHA_2_DS_2_-VASc ≥ 2, we find the same differences as in the overall group: it seems that there is higher mortality among patient living in urban environmental (17.2% versus 6.4%) but without statistical significance, among those with ≥ 3 CVCs before the AF diagnosis (P = 0.013), and those with antiplatelet treatment versus antithrombotic (P = 0.025).

The lineal regression results found as associated variables to more risk of mortality are: age, score CHA_2_DS_2_-VASc and the only treatment with antiplatelet agents. Any other variable was associated to better survival or mortality. In the Cox regression, after adjusting by age, gender, number of CVCs before and after AF diagnosis, antithrombotic treatment, antiplatelet treatment, following by cardiologist, and other specific treatments, the only variable with protector value on the mortality was the antithrombotic treatment (OR: 0.344; 95% CI: 0.163 - 0.728).

## Discussion

AF is associated with significant cardiovascular morbidity before and after its diagnosis, mainly ischemic heart disease, ischemic stroke and congestive heart failure and requires chronic use of medication, but the relationship between AF and mortality keeps less clear. Prior myocardial infarction and the heart failure have been identified as one of the strongest independent predictor of cardiac death [4]. The average age is not different from the global population in Spain [19] and Catalonia [20] where the life expectancy at birth is 82 years and at age 60 is 25 years. So far we could not demonstrate a higher mortality [21] nor differences in the mortality by gender. Maybe we should research a renewed focus on evaluation and management of CV comorbidities and risk factors as the more effective at reducing overall mortality in these patients.

In its evolution, we could describe two different paths: previous and after AF. Around 6 years prior to the AF, the ischemic cardiopathy and the ischemic stroke are the most frequent and its incidence is higher significantly among men and associated to increased risk of mortality if the number of diagnosed CVCs is ≥ 3. However, there are also particularities that happen after diagnosing AF; in this way in an average of 4 years after the congestive heart failure, there exist the main CVC and significantly higher among women and their coexistence is associated with adverse prognosis [20]. On the one hand, it seems that there could be a trigger on underlying disease of the cardiovascular system which would mean the initiation and maintenance of AF, but its occurrence cannot be predicted. On the other hand, AF would be one condition which may be worse than the prognosis and more frequently among women though we have not evidenced higher global mortality among them [21].

Eventually, there would be other subset of patients without evidence of CVCs and different epidemiological and mortality risk. We should think about if we may be able to improve the prognostic and lower the mortality by a more intensive treatment and if the factors like age, score CHA_2_DS_2_-VASc or antithrombotic treatment may be helpful in identifying “at-risk” patients and guiding a therapy. The complex relationship between AF and its most frequent associated complications will continue to increase in the setting of an aging global population and we should improve management strategies. These decisions involve address in the key topics of heart rate control, heart rhythm control, and stroke prevention.

In reference to patient’s group with AF but without diagnosed CVCs, we know the AF sometimes develops in a subset of young patients with no evidence of associated cardiopulmonary or other comorbidities (including hypertension), and it has been referred to as “lone AF”, which generally has a favorable prognosis [5, 21-26]. Our subset of patients without CVCs would be similar to what has been defined as “lone AF” between 1.6% and 30%, depending on the patients’ age and criteria used in the study [5, 24, 27] but in the item of the hypertension prevalence that in our study was 62%. We have to consider that the term “lone AF” is still kept being an exclusion diagnosis [7]. Moreover, their age is lower than the remaining and their prognosis is better according to their survival curve [5, 6, 10, 11], perhaps for the simple fact of not having CVCs these days. In some studies, lone AF patients may have a similar risk of thromboembolism, CHF and mortality as the general population [5, 6, 23]. However, other studies suggest different risks. Our study suggests that lone AF could carry a better prognosis when is treated with antithrombotic treatment, regarding thromboembolism and mortality [5] though its evolution during the follow-up will be significantly influenced by ageing and development of comorbidities. Nevertheless, it is interesting because the lone AF and the paroxysmal episodes could be reasons which would explain, at least relatively, the AF percentage non-diagnosed among general people [1]. To date, genetic studies have revealed diverse mechanisms of susceptibility to AF. A genetic contribution to AF was recognized nearly 70 years ago. Since then, the familial aggregation of lone AF has been increasingly reported and the heritability of AF in the general population has been documented in several population-based studies [28-31]. Under this evidence, should we make an ECG to aquattancies or prolonged continuous cardiac monitoring [32] to prevent, diagnose early the AF or prevent its potential complications?

In spite of the great impact of AF as a health problem, the knowledge about the prediction and prevention of complications is complicated. If we make an evaluation of the AF coverage with specific treatment, especially in the subset with score CHA_2_DS_2_-VASc ≥ 2 and the overall group, the global result is not different because this group is the 95.6% of the patient group with AF diagnosis.

Rate control approach remains the standard therapy for AF in heart failure because current strategies at rhythm control have so far failed to positively impact mortality and morbidity [23]. In patients with AF and heart failure, the use of beta-blockers [33] and ACE [34] was associated with a 42% reduction in mortality. So given CHF the CVC is more frequent after AF diagnosis and the strongest independent predictor of death in this population [4], starting a treatment with ACE or beta-blockers at the moment of its diagnosis and, previously, the clinical evidence of the congestive heart failure would allow a research about if it would be a reduction in its incidence. Several studies have reported the efficacy of ACE inhibitors in preventing AF and a meta-analysis [35] found that the greatest reduction in AF was in patients with heart failure. In this way, new biomarkers [36] may help to understand the mechanisms of subclinical AF and signal the likelihood of disease progression. However, the ability of ACE inhibitors or ARBs to prevent cardiac failure associated with AF, when used in its diagnostic, has yet to be evaluated.

Eventually, when there is AF, correct OAC treatment is a must as several randomized trials have demonstrated, given that a suboptimal anticoagulation has been associated with poor outcomes in terms of mortality [37] and there is a greatest likelihood of a lower TTR [38] when was associated with heart failure, our results also showed that the survival chance with a diagnosis of CHF was lower (0.69 ± DE 0.09) than when there is no CHF present, but the mean TTR had not different distribution in patients with CHF.

### Conclusions

AF is associated with significant cardiovascular morbidities mainly ischemic heart disease, ischemic stroke and congestive heart failure and requires chronic use of medication, but the relationship between AF and mortality keeps less clear. The average age is not different from the global population in Spain and Catalonia. The significantly differences in mortality between the group with/without statins in the patients with CVCs before AF and the anticoagulant treatment as the only variable with protector value on the mortality (OR: 0.344; 95% CI: 0.163 - 0.728) reinforce strongly to use these medications. The higher mortality and the higher incidence of CHF among women should advice institute adequate diagnosis, therapy and prevent complications related to the congestive heart failure.

## Trial Registration

The research protocol was evaluated and approved by the Clinical Research Ethics Committee of the Primary Care Research Institute, IDIAP Jordi Gol, Barcelona June 12, 2009 (Ref: 5011/011) and by the Clinical Research Ethics Committee of the Hospital University of Tarragona Joan XXIII (Ref: 35/2011).

## Abbreviations

ACE: angiotensin-coverting-enzime
AF: atrial fibrillation
ARBs: angiotensin receptor blockers
CHA_2_DS_2_-VASc: (C)ongestive heart failure (or left ventricular systolic dysfunction), (H)ypertension, blood pressure consistently above 140/90 mmHg (or treated hypertension on medication), (A)ge ≥ 75 years, (D)iabetes mellitus prior, (S)troke or TIA or thromboembolism, (V)ascular disease (previous MI, peripheral arterial disease or aortic plaque), (A)ge 65-74 years, (S)ex category (female gender)
CHF: cardiac heart failure
CVCs: cardiovascular comorbidities
OAC: oral anticoagulants
TTR: time in therapeutic range
